# Investigating the Material Properties and Microstructural Changes of Fused Filament Fabricated PLA and Tough-PLA Parts

**DOI:** 10.3390/polym13091487

**Published:** 2021-05-06

**Authors:** Nida Naveed

**Affiliations:** Faculty of Technology, University of Sunderland, Sunderland SR6 0DD, UK; nida.naveed@sunderland.ac.uk

**Keywords:** 3D printer, PLA, tough PLA, fused deposition modelling (FDM), additive manufacturing (AM), microstructural analysis, raster angle

## Abstract

Fused deposition modelling (FDM) is a popular but complex additive manufacturing process that works with many process parameters which are crucial to investigate. In this study, 3D parts were fabricated by placing each filament layer in opposite direction to the others; for this, two combinations of raster angles, (45° −45°) and (0° 90°), along with three different infill speeds were used. In this study, two 3D printing material types—Polylactic Acid (PLA) and tough-PLA were used. The material properties of each 3D part were investigated to identify the best combination of these parameters. A microstructural analysis was also performed on outer and inner surfaces along with fracture interface of the parts after tensile testing using a scanning-electron-microscopy (SEM) to explain material failure modes and reasons. The results suggest that for both the material types, a raster angle of 45° −45° produces stronger parts than to a raster angle of 0° 90°. This study also suggests that a slow infill speed improves tensile properties by providing a better inner-connection between two contiguous roasters. Thus, the detailed analysis of microstructural defects correlated with tensile test results provides insight into the optimisation of raster angle and infill speed, and scope for improvement of mechanical properties.

## 1. Introduction

Fused deposition modelling (FDM) is referred to as a modern additive manufacturing (AM) technique that is most used for 3D printing. Three-dimensional printing is the process of making three-dimensional objects by depositing multiple thin layers using a wide variety of materials such as plastic, glass, ceramics and metals. It involves the design of a digital model of a 3D part using computer-aided design (CAD) software and uploading it into the 3D printer machine to transform it into physical form [1,2,3]. This technology can be referred to as the fast-manufacturing process and it is most significant for small industries. It has the capability to speed up the whole production process compared to conventional manufacturing techniques [4].

In the AM process, both low-melting-point materials such as plastics, resins and polymers and high-melting-point materials such as alloys, metals and ceramics can be used. In addition, some novel materials such as composites, multiple materials, metamaterial and functionally gradient materials are under investigation to be used for the AM process [5]. The most common thermoplastic materials that are used in the FDM process to create 3D parts are polyamide (PA), polylactide (PLA), polycarbonate (PC), tough polylactide (tough PLA) and acrylonitrile butadiene styrene (ABS) [6,7]. PLA material has gained wider acceptance within 3D printing due to its good mechanical properties, high reliability, low cost, good dimensional accuracy and surface finish as well as that it is prepared using renewable products [8,9]. PLA is a polymer called polylactic acid which is made from organic and renewable resources such as potato starch and sugar cane. It is easy to print with and strong but more brittle compared to other 3D printer materials such as ABS [10]. It has a low coefficient of thermal expansion which limits its applications in which the printed part is exposed to temperatures higher than 50 °C [11,12]. Tough PLA is the same as PLA with a better toughness comparable to ABS. It has a stronger impact resistance than PLA. Tough PLA bends before it breaks, which makes it well suitable for many engineering applications, especially where high-wear and impact resistance are required [13]. Similarly to standard PLA, it has a low coefficient of thermal expansion so printed parts should not be exposed to temperatures above 60 °C [14]. Tough PLA has some unique material properties such as better toughness and stronger impact resistance that are important to investigate to enhance its engineering applications.

There are many applications of the PLA material in the medical field such as for the manufacturing of bioresorbable stents and sutures. Jörgen S. B. et al. investigated the microstructural states and the mechanical behaviours of various bioresorbable polylactide (PLA) devices for medical applications [15]. In another interesting study, the surfaces of polylactic acid (PLA) nonwovens, which are used in medical dressings, were treated with magnetron sputtering to deposit nano-structured silver films. In this study, the effects of different thicknesses of nano-structured sliver film coatings were examined on the antibacterial property of nonwovens and it was found that it is very helpful to reduce the antibacterial property of polylactic acid (PLA) nonwovens [16]. PLA material can also be used in other pharmaceutical and medical fields, such as tissue engineering, to develop 3D structures including scaffolds, artificial skins, and bone fracture internal fixation devices [17,18,19].

Fused deposition modelling (FDM) is one of the most promising AM techniques for 3D printing. However, FDM is a complex process which is based on many parameters [20,21]. A critical literature review suggests that the part quality is the function of various process parameters and can be significantly affected by any small change of these process parameters. Since it is essential to gain the desirable material properties of the printed part from a technological point of view to improve its serviceability, it is crucial to investigate the influence of various FDM process parameters on mechanical properties of a printed part so that the best quality of the part through selection of the optimum settings can be obtained.

In the available literature, many studies have been conducted on 3D-printing process parameters which have discussed their influence on material properties and its behaviour. In one of these studies, the mechanical strength and material porosity were found to be significantly affected by the two main process parameters: raster width and air gaps [22]. Another study pointed out the five process parameters (layer thickness, orientation, raster angle, raster width and air gap) as important parameters that have a large influence on the tensile, flexure and impact strength of a 3D-printed part made up of ABS material [23]. In another study, two raster orientations, one in cross (0°/90°) and the other in crisscross (45°/−45°) direction, were investigated using ABS material and it was found that crisscross (45°/−45°) orientations provide better material strength compared to the other orientation [24]. In another previous study, the effect of layer height, infill density, and layer orientation on the mechanical properties of PLA and ABS were investigated and it was identified that PLA is more suitable for the use of 3D printing material [25]. Letcher et al. [26] investigated the three raster orientations 0°, 45° and 90° using PLA material and identified that the 45° raster orientation provided the strongest material behaviour. In another study, the five different raster angles (0°, 30°, 45°, 60° and 90°) were used to fabricate 3D parts using PLA, and their tensile properties were investigated. The study identified that the roaster angles 45° and 90° produced specimens with better strength. It also identified that there are several defects in 3D-printed parts at micro level that have a large impact on the mechanical properties of a 3D-printed part [27].

It is vital to explore the effects of different raster angle positions with the combination of other 3D-printing process parameters on microstructural changes that directly manipulate the material strength. Raster orientation is one of the most important parameters of the FDM 3D-printing process. Raster orientation-part build orientation refers to the inclination of a part in a build platform with respect to *x*, *y* and *z* axis. The *x* and *y* axes represent the plane parallel to the build platform and the *z* axis represents the vertical plane along the direction of the part build [23]. Infill speed is another very important parameter of the FDM 3D-printing process; it is the speed of the nozzle traveling relative to the print bed. This controls the volume of the extruded filament and cross-sectional geometry of the sample [28]. In this study, two part-depositing parameters—raster orientation and infill speed—were studied. Two raster orientation angles (45°, −45° and 0°, 90°)—where 0° indicates the transvers direction, 90° is the axial direction along the specimen length in the corresponding printing orientation, 45° is the 45° inclination of a part in a build platform with respect to the *x*-axis and −45° is the same inclination of a part as 45° but in the opposite direction—were used with three different infill speeds to fabricate 3D samples. In this study, the effects of these process parameters were investigated with in relation to the two most commonly used AM 3D printing materials PLA and tough PLA. A tensile test was performed for each 3D-printed specimen and the effects of these process parameters on tensile strength were investigated. A micro-structural analysis was also performed on the fracture interface of the specimens after tensile testing using a scanning electron microscope to explain material failure modes and reasons for them. In this study, micro-level structural changes on the outer and inner surfaces of the 3D samples were also examined in detail.

## 2. 3D printing Material Specification

For this study, two 3D printing material types, standard PLA and Tough PLA, are used. Table 1 and Table 2 show the specifications and mechanical properties of PLA and Tough PLA filaments [29].

## 3. Experimental Procedures

For this study, the experimental work consisted of preparation of the standardised tensile test specimens using a 3D printer, testing of 3D-printed specimens and detailed microstructural analysis of the outer and inner surfaces of the 3D-printed specimens. Information about each step is provided in detail below.

### 3.1. The 3D-Printing Process

**Figure 1:**
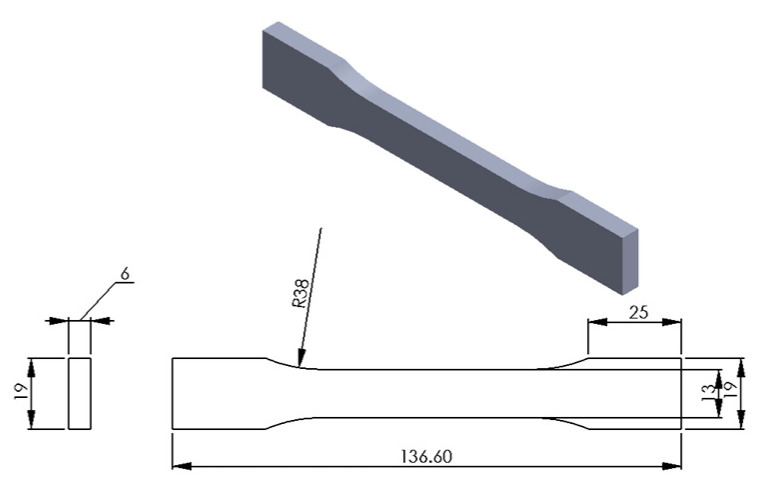
Geometry of the tensile test specimen according to ASTM D638 standard (all dimensions are in mm).

**Figure 2:**
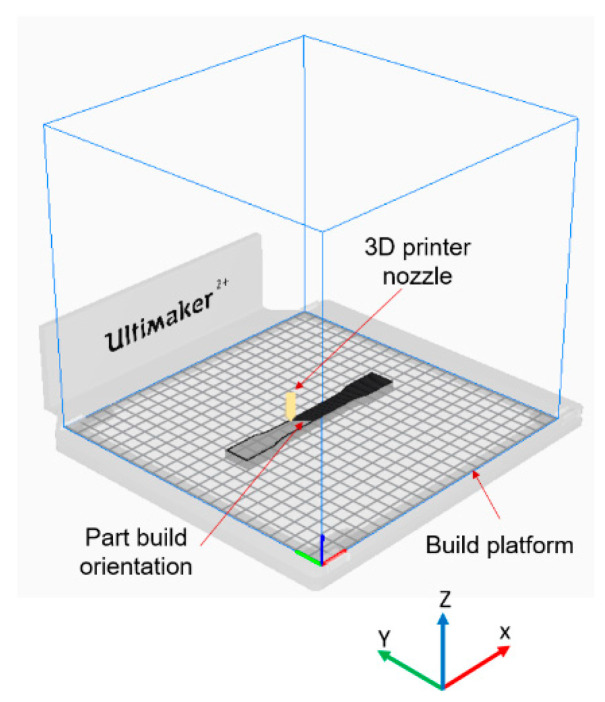
Tensile specimen on 3D printer bed—image captured from 3D printer software Cura 4.3.0.

**Figure 3:**
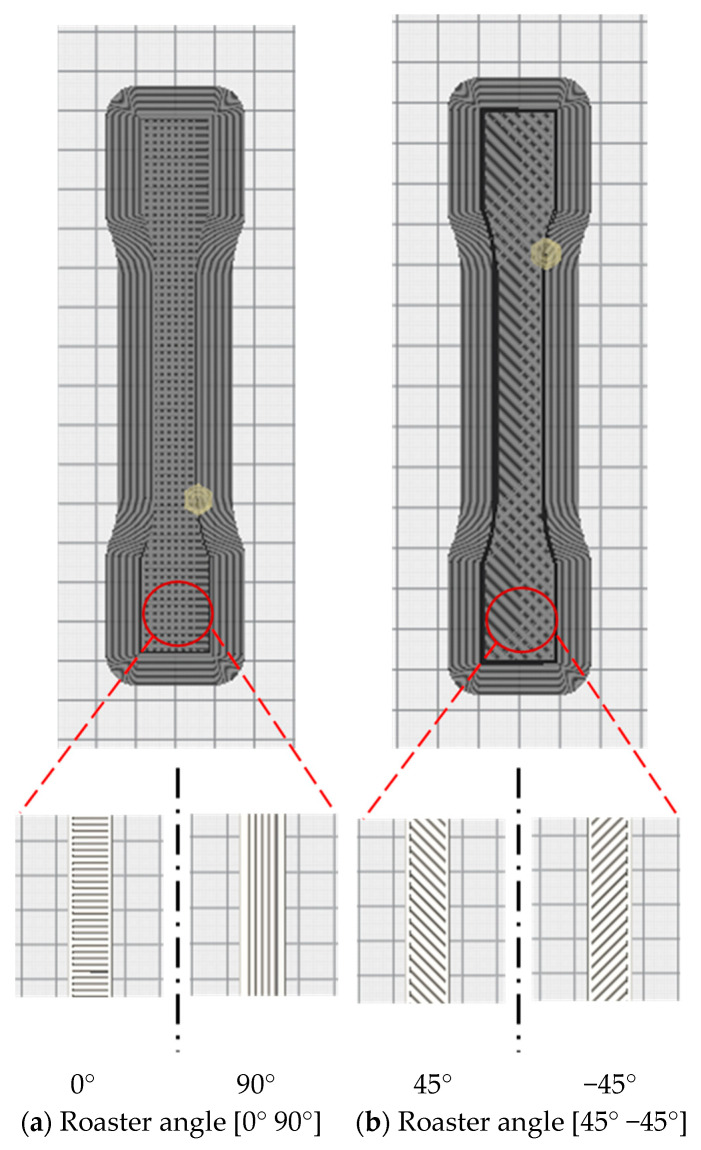
(**a**) Images for the roaster angle (0° 90°). (**b**) Images for the roaster angle (45° −45°). All images captured from Ultimaker 3D printer software Cura 4.3.0.

Firstly, the test specimens were prepared using 3D printer according to the required specifications. For that, the 3D CAD SolidWorks 2019 software was used to develop three-dimensional virtual geometry of the test specimen. The geometry of the specimen was developed according to the ASTM D638 standard tensile test specimen [30], as shown in Figure 1. After developing the virtual specimen geometry, the SolidWorks file was converted into STL format. The 3D printer accepts this format to physically print the specimen. Then, the STL file was transferred to the 3D-printer-controlled computer. The 3D printer Ultimaker 2 was employed to print the specimens as it is a powerful, simple and reliable 3D printer technology. It operates through a touchscreen interface that guides and displays detailed status information. Ultimaker Cura 4.3.0—3D printing software was used to prepare the design that was developed in SolidWorks for 3D printing. Figure 2 shows the image of the tensile specimen on the 3D-printer bed captured by the software. This software can manage and monitor the print progress and maintenance schedules, queue prints and manage different software designs. For printing, custom printing settings were used to give in-depth control to the printing software, which allows the printer to specify printing parameter settings such as different raster orientations and infill speeds. This 3D printer extruded the PLA at 200 °C and Tough PLA at 225 °C with the heated bed surface at 60 °C to prevent warping on the first layer of the specimen. The specimens were printed with two raster orientation angles (45° −45°) and (0° 90°) (as shown in Figure 3) at three different infill speeds, 35, 50 and 65 mm/s, respectively. An infill density of 100% and an infill later thickness of 0.1 mm were used for each raster orientation. There were three identical tensile samples printed with each combination of the selected parameters and altogether, 36 samples were printed. Table 3 shows the details of the selected process parameters that were used for 3D printing. All the specimens were built with 750g spools of PLA and Tough PLA materials. Each specimen takes approximately 3 h 30 min to print for material with a mass of 17 g. This printer begins printing first the build of a thin layer of base support and then it builds an outer layer base and fills the specimen with a specific raster orientation.

### 3.2. Tensile Testing for 3D-Printed Specimens

All the 3D-printed specimens were tested according to the ASTM D638 standard testing method for tensile testing of plastics material [30]. A universal tensile testing machine was used to conduct the tensile test. The specimens were tested at a speed of 5 mm/min. Specimens were set for this test machine using the desktop testXpert2 software. This software also collects the displacement (mm) and force (*n*) values for each test in order to obtain stress–strain curves.

### 3.3. Sample Preparation for Microstructural Examination

The material strength of any material is directly related to its microstructure. Therefore, microstructure examination of the fractured surface is vital to understand the failure behaviour and failure mode. In this study, the fractured interface of the tensile specimens after tensile testing were analysed. In addition, the outer surfaces of the tensile specimens were also examined to identify any defect formed during 3D printing. In this study, a scanning electron microscope (SEM) S-3000N Hitachi was used at an acceleration of 5 KV in the high vacuumed mode to examine the microstructure of the material. In order to prepare the samples for SEM analysis, the specimens were broken into small pieces along the fractured surfaces. Then, the samples were vacuum sputter-coated with a thin layer of gold-palladium alloy to eliminate the charging effects of the surface. This thin layer coating is important as it provides a homogeneous surface for analysis and imaging [31].

## 4. Results and Discussion

### 4.1. Tensile Test

Three identical tensile samples were tested for each combination of the 3D-printing process parameters selected and overall, 36 samples were tested. A summary of all the tensile test results for PLA and tough PLA is provided in Table 4 and Table 5.

Table 4 and Table 5 show the tensile test results that demonstrate the effects of raster orientations and infill speeds on tensile properties, including ultimate strength, modulus of elasticity and elongation at break for both PLA and tough PLA. For both materials—PLA and tough PLA, the 45° −45° raster orientation with a 35-mm/s infill speed produced strong specimens with average ultimate tensile strengths of 64.57 MPa and 53.60 MPa, and highest elongations of 6.6% and 6.8%, respectively. The 0° 90° raster orientations with the same 35-mm/s infill speed produced specimens with average ultimate tensile strengths of 59.17 MPa and 46.93 MPa (8% and 12% less than the strength of the specimens produced with the 45° −45° raster orientation with the same speed). Overall, the 45° −45° raster orientation produced the strongest specimen for both materials and for all three infill speeds. The infill speed of 35 mm/s produced the strongest specimen. The value of the ultimate strength decreases as the infill speed increases. A possible reason for this is that the increase in infill speed reduces the deposition time, which results in less interaction and lower inner-connection for the creation of a bond between two contiguous roasters and causes a decrease in tensile properties.

The standard PLA produced strong specimens compared to the tough PLA for all combination of the process parameters of 3D printing that were used in this study. However, tough PLA produced better elongation than standard PLA. These results were also compared with previous research [27] in which single 45° and 90° raster orientations were used with an infill speed of 70 mm/s for the PLA material type. These results indicate that the ultimate tensile strength of the specimen improved by 14% and similarly, the percentage of the elongation improved by 36% using a 45° −45° raster orientation and a 35 mm/s infill speed. Overall, these results show that the position of the raster and the infill speed are the most important parameters for 3D printing and have a vital role in specimen strength. It can also be concluded that in the present study, the roaster angle of 45° −45° and an infill of 35 mm/s are the most suitable values for these parameters to achieve a high tensile strength value for standard PLA and tough PLA.

**Figure 4:**
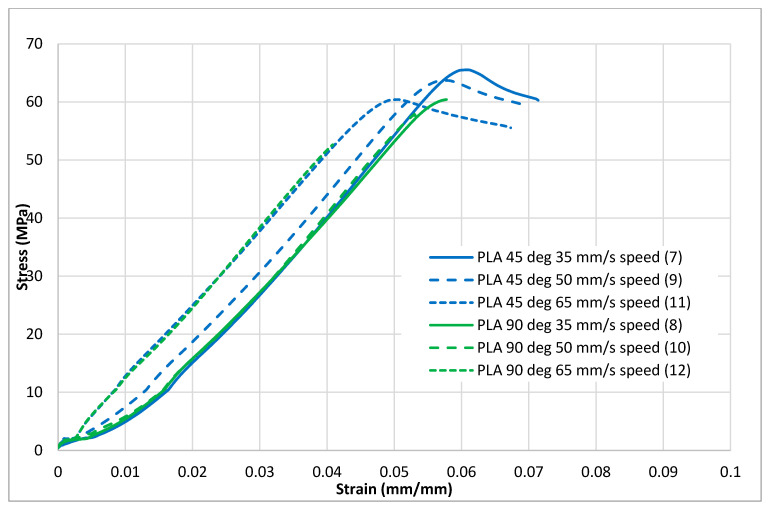
Tensile test results (stress–strain curves) for the standard PLA material.

**Figure 5:**
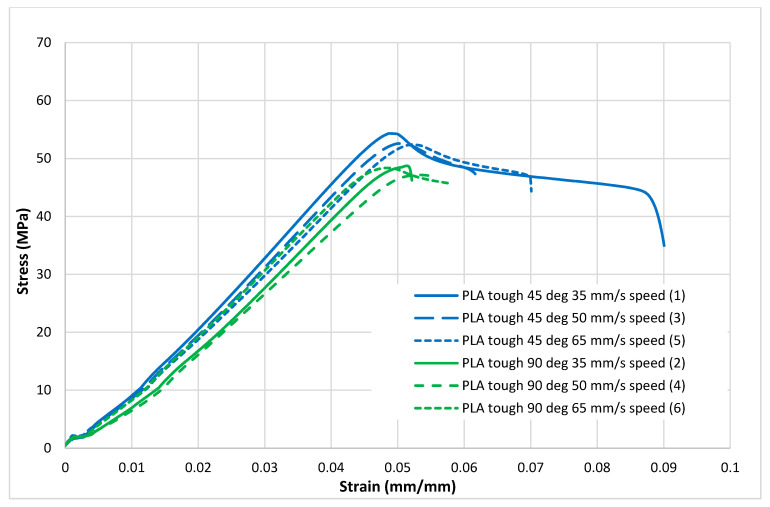
Tensile test results (stress–strain curves) for the tough PLA material.

**Figure 6:**
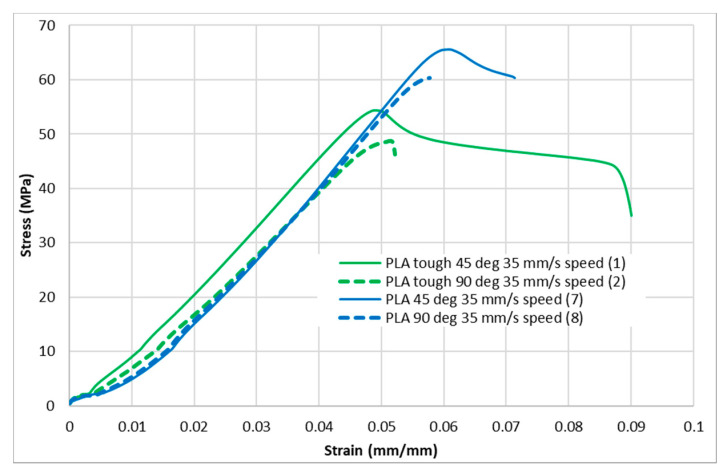
Stress–strain curves for PLA and tough PLA at a 35 mm/s infill speed.

**Figure 7:**
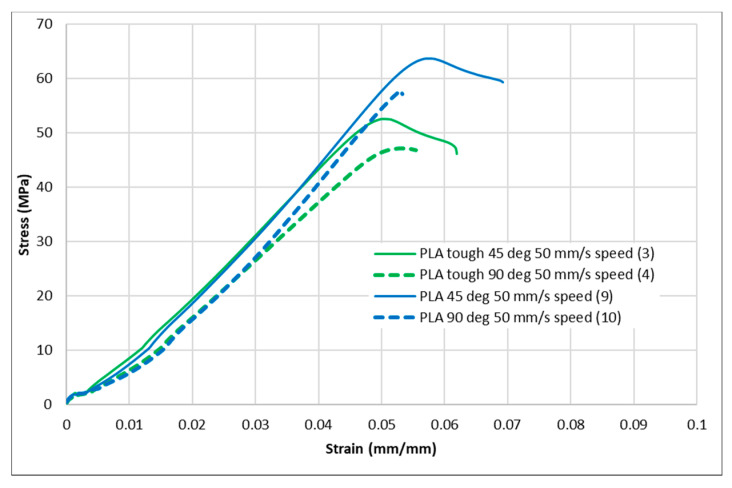
Stress–strain curves for PLA and tough PLA at a 50 mm/s infill speed.

**Figure 8:**
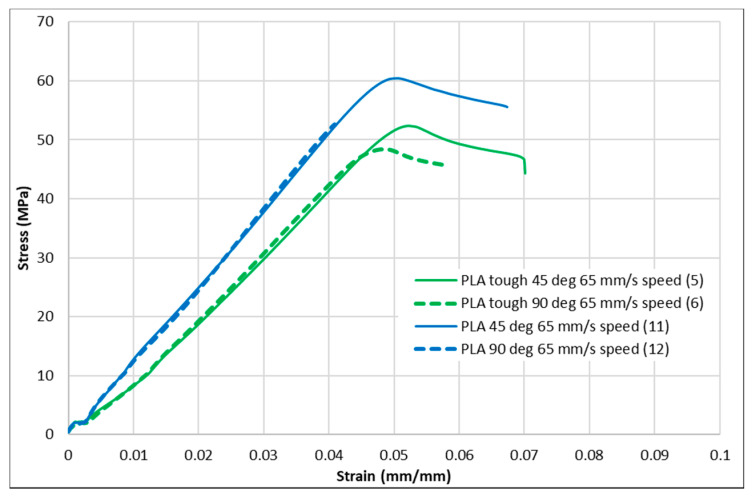
Stress–strain curves for PLA and tough PLA at a 65 mm/s infill speed.

Figure 4, Figure 5, Figure 6, Figure 7 and Figure 8 show the stress–strain curves for the specimens that were printed with two raster orientations and three infill speeds using PLA and tough PLA materials. The effect of different combinations of the 3D-printing process parameters on material behaviours such as strength, toughness, stiffness and hardness can be identified using these curves. Figure 4, Figure 5, Figure 6, Figure 7 and Figure 8 show that the PLA material achieved high stress peaks compared to tough PLA and formed strong specimens. These stress peaks decline as the raster orientation shifts from 45° −45° to 0° 90° and infill speed increases from 35 mm/s to 65 mm/s. Another important property of the material is toughness. Toughness is the measure of a material’s ability to absorb energy before it fails, and it can be measured by the area under the stress–strain curve [32]. In terms of identifying the toughness behaviour of these specimens, Figure 5 shows that the specimen that was printed using the material with a tough PLA with the process parameters of a raster orientation of 45° −45° and an infill speed of 35 mm/s presents the highest toughness behaviour of all the specimens. It is also evident that the specimen toughness decreases as the infill speed increases. The specimens that were fabricated using PLA and tough PLA with raster orientations of 45° −45° and 0° 90° show similar initial slopes. However, after examining the whole curve, it can be identified that the specimens that were printed with a raster orientation of 0° 90° show simply elastic deformation, which signifies stiff material behaviour. However, the specimens that were fabricated with the raster orientations 45° −45° show both elastic and plastic deformation and represent hard material behaviour. These phenomena were further explored by fracture interface examinations on these tensile specimens.

### 4.2. Outer Surface Observations of Tensile Specimens

**Figure 9:**
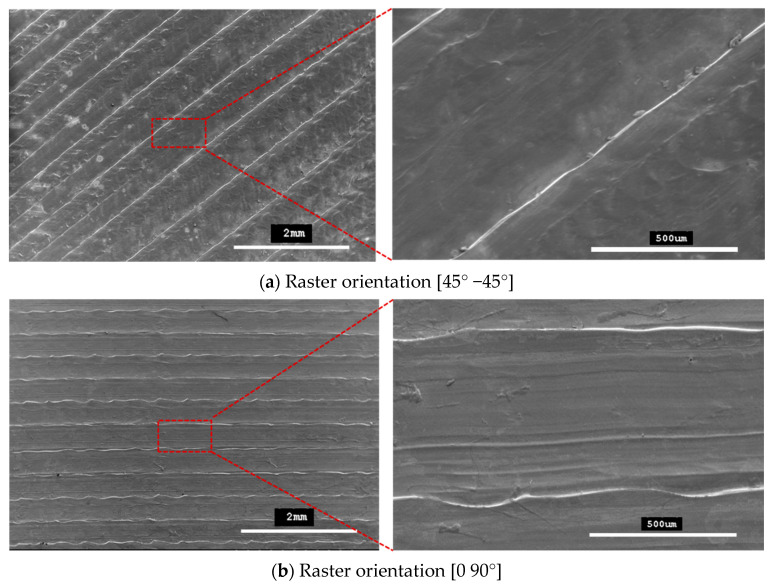
SEM micrographs showing printing patterns on specimens fabricated with (**a**) raster orientations (45° −45°) and (**b**) raster orientations (0° 90°).

Figure 9 shows SEM micrographs of the outer top surfaces of the tensile specimens. These micrographs confirm that printed patterns formed with 45° −45° and 0 90° raster orientations.

### 4.3. 3D Printing Defects

Any defects, such as openings, cracks, voids and air gaps, in 3D-printed parts adversely affect the material properties of parts. Therefore, these defects must be avoided in the final finished form of a 3D-printed part. In this study, SEM microstructure images were used to identify these defects in 3D-printed specimens that were fabricated using two raster orientations and three different infill speeds.

**Figure 10:**
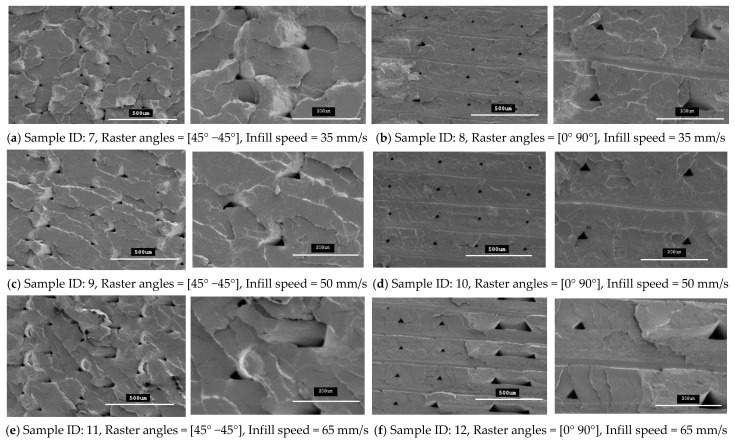
SEM images of the fractured interface of tensile specimens fabricated with two different raster orientations (45° −45° and 0 90°) with three infill speeds for the PLA material type.

**Figure 11:**
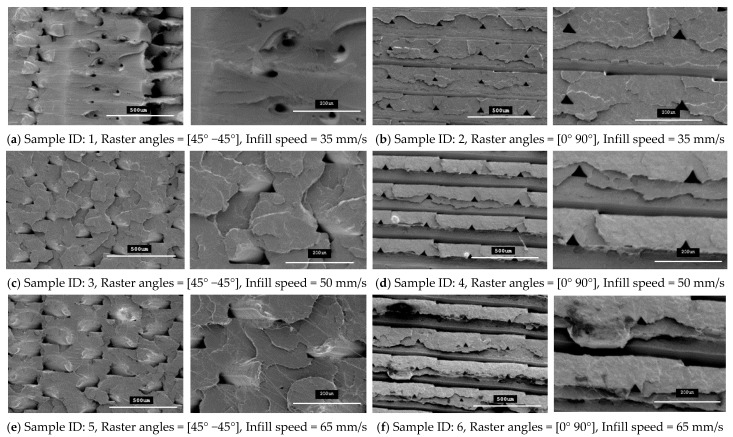
SEM images of the fractured interface of tensile specimens fabricated with two different raster orientations (45° −45° and (0 90°) with three infill speeds for the tough PLA material type.

The specimens that were fabricated with raster angles of 45° −45° and 0° 90° formed compact outer surfaces and any defect cannot be seen on the outer surfaces of the specimens, as reported previously with other roaster angles [26]. Thus, an overall better surface finish was achieved with these two raster angles. However, some inner defects can still be seen on the fracture interfaces for these specimens. In this study, the fracture interface of all the tensile specimens that were printed with twelve different combinations of 3D-printing parameters were investigated to explore the internal quality of the 3D-printed specimens. Figure 10 and Figure 11 show SEM images for all the fractured interfaces that formed after tensile testing. These images identify several problems in the placement of layers in the 3D-printing process.

These images show that both raster orientations formed a series of triangular voids between two layers all along the thickness of the specimen and this occurred in both PLA and tough PLA materials for all twelve combination of process parameters. These voids are most likely related to the deposition angles of the filament (roaster angles) that cause these cavities between two contiguous roasters. Table 6 shows the average area of these triangular voids that were captured during SEM scanning. The area of the voids was calculated by measuring the size of the different voids in the SEM images and then the area was calculated according to the geometry of the voids. Larger voids were formed in the tough PLA materials compared to the standard PLA materials for almost all the specimens. In addition, for tough PLA, larger voids were formed for a raster angle of 45° −45° compared to a raster angle of 0° 90°. For a raster angle of 45° −45°, the size of the voids increases when the infill speed increases. However, for a raster angle 0° 90°, the infill speed had an effect on the size of the voids in the standard PLA. In the tough PLA specimens, triangular-shaped voids can be seen very clearly as well as air gaps between two adjacent layers in the longitudinal direction (parallel to the layer). This defect is unique to tough PLA and it does not appear in standard PLA material. These defects directly affected the specimen’s material strength and formed weaker specimens. Similarly, in this study, the specimens fabricated with tough PLA showed lower tensile strength compared to the specimens fabricated with the standard PLA. A possible reason for the poor performance of the tough PLA is that a different nozzle temperature was used for each material; PLA was extruded at 200 °C and Tough PLA at 225 °C, which caused different heating and cooling rates during the printing process. The 225 °C temperature could form a more complete merge of the roaster but more degradation was generated inside the specimens at micron-level, which led to a decrease in tensile properties [23,27]. The specimen that was fabricated using the standard PLA with a raster angle of 45° −45° and an infill speed of 35 mm/sec shows better results with the highest material strength of 64.57 MPa and less fracture interface defects. It is evident that the strength of these specimens can be further improved by avoiding these printing problems and defects. Thus, raster orientation and infill speed have an effect on 3D-printed part strength. Suitable selection of these process parameters can increase the strength and precision of the part by reducing the gaps between the deposited layers.

### 4.4. Fracture Interface Observations After Tensile Test

Figure 10 and Figure 11 show SEM micrographs of the fracture interface of the tensile specimens that were fabricated with two raster angles and three different infill speeds using PLA and tough PLA materials, respectively. The specimens that were printed with a raster angle of 45° −45° show the most roughly fractured surfaces compared to the specimens that were printed with a raster angle of 0° 90°. In Figure 10 and Figure 11a,c,e, the specimens that were fabricated with a raster angle of 45° −45° fractured due to material failing during tensile loading; therefore, larger tensile strength and tensile modules were achieved. As shown in Figure 10 and Figure 11b,d,f, the specimens that were fabricated with raster angles of 0° 90° got ruptured along the 0° roaster layers and formed smooth fractured surfaces. The specimens that were fabricated with this raster orientation failed due to breakdown in the interfacial adhesion between two adjacent layers. This raster orientation does not allow effective transfer of the tensile load from one layer to another layer, thereby the specimen does not resist the full tensile load and breaks before the material reaches its tensile strength. Therefore, the specimens that were printed with a raster angle of 45° −45° show better strength and more confine fracture surfaces compared to the specimens that were fabricated with a roaster angle of 0° 90°.

## 5. Conclusions

In this study, twelve combinations of 3D process parameters of two raster angles and three infill speeds were used to fabricate 3D-printed specimens using standard PLA and tough PLA material types. The following conclusions can be drawn from this study.

Raster orientation and infill speed are the most important parameters for the 3D printing-process and they have a vital role in specimen strength, printing quality and surface finish. Standard PLA produced stronger specimens than tough PLA. However, tough PLA gave better elongation than standard PLA. For both the materials, the raster orientation of 45° −45° with a low infill speed showed a high tensile strength and high elongation at break than the raster orientation of 0° 90°. A low infill speed increases the strength and toughness of the specimens by providing a longer deposition time, which allows adequate time to create a bond between two contiguous roasters. The specimens that were fabricated using standard PLA and tough PLA with raster orientations of 45° −45° and 0° 90° show a similar initial slope in the strain–stress curve. However, after examining the whole curve, it can be identified that the raster orientation of 0° 90° simply shows elastic deformation which signifies stiff material behaviour. Nevertheless, the raster orientation of 45° −45° shows both elastic and plastic deformation and represents hard material behaviour. The raster angle of 45° −45° produces specimen with better strength and more confined fracture surfaces compared to the roaster angle of 0° 90°. The roaster angles of 45° −45° and 0 90° formed compact and smooth outer surfaces for both the material types; those outer surfaces are free from any defects such as voids, air gaps and cracks. The micro-level observations of the fracture interfaces (inner surfaces) show a series of air gaps and voids. These microstructural defects are directly correlated with the tensile strength results. The strength and accuracy of 3D-printed parts can be further improved by avoiding these printing problems and defects.

## Figures and Tables

**Table 1:** PLA and Tough PLA filaments specifications.

Material Type	PLA	Tough PLA
Diameter (mm)	2.85 ± 0.10	2.85 ± 0.10
Max roundness deviation (mm)	0.10	0.05
Net filament weight (g)	750	750
Filament length (m)	95	96
Colour	black	white

**Table 2:** Mechanical properties of PLA and tough PLA filaments.

	PLA	Tough PLA
Tensile modulus (GPa)	2.34	1.82
Tensile stress at break (MPa)	45.6	37
Elongation at break (%)	5.2	3.1

**Table 3:** The process parameters used to fabricate the samples.

**PLA**	**Tough PLA**	**Raster Angles**	Infill Speed (mm/s)
**Sample ID**			
7	1	45° −45°	35
8	2	0° 90°	35
9	3	45° −45°	50
10	4	0° 90°	50
11	5	45° −45°	65
12	6	0° 90°	65

**Table 4:** Summary of the tensile test results for the PLA material type.

Sample ID	Raster Orientation Angle (degree)	Infill Speed (mm/s)	Elongation at Break (%)	Modulus Elasticity (GPa)	Ultimate Stress (MPa)
7	[45° −45°]	35	6.62 ± 0.98	0.81 ± 0.06	64.57 ± 0.95
9	[45° −45°]	50	6.67 ± 0.21	0.80 ± 0.11	62.13 ± 2.38
11	[45° −45°]	65	6.25 ± 0.34	1.07 ± 0.25	58.77 ± 1.42
8	[0° 90°]	35	5.82 ± 0.61	0.81 ± 0.06	59.17 ± 1.25
10	[0° 90°]	50	4.85 ± 0.45	0.79 ± 0.03	52.90 ± 8.23
12	[0° 90°]	65	4.04 ± 0.04	1.22 ± 0.02	51.67 ± 0.95

**Table 5:** Summary of the tensile test results for the tough PLA material type.

Sample ID	Raster Orientation Angle (degree)	Infill Speed (mm/s)	Elongation at Break (%)	Modulus Elasticity (GPa)	Ultimate Stress (MPa)
1	[45° −45°]	35	6.88 ± 1.85	1.00 ± 0.09	53.60 ± 0.82
3	[45° −45°]	50	6.02 ± 0.58	0.82 ± 0.06	51.20 ± 1.22
5	[45° −45°]	65	6.80 ± 1.29	0.82	50.67 ± 1.50
2	[0° 90°]	35	4.63 ± 0.41	0.87 ± 0.09	46.93 ± 3.79
4	[0° 90°]	50	4.94 ± 0.43	0.78 ± 0.03	46.33 ± 0.76
6	[0° 90°]	65	4.78 ± 0.79	0.83 ± 0.05	45.50 ± 2.59

**Table 6:** Averaged area of voids that were captured during SEM scanning.

Sample ID PLA/Tough PLA	Raster Orientation Angle (degree)	Infill Speed (mm/s)	Voids Area (µm^2^) PLA	Voids Area (µm^2^) Tough PLA
7/1	[45° −45°]	35	143	1434
9/3	[45° −45°]	50	236	2626
11/5	[45° −45°]	65	1054	8504
8/2	[0° 90°]	35	727	738
10/4	[0° 90°]	50	383	1117
12/6	[0° 90°]	65	822	366

## Data Availability

The data presented in this study are available on request from the corresponding author.
